# Delirium screening in the intensive care unit using emerging QEEG techniques: A pilot study

**DOI:** 10.3934/Neuroscience.2020001

**Published:** 2020-01-13

**Authors:** Andrew Hunter, Barry Crouch, Nigel Webster, Bettina Platt

**Affiliations:** Institute of Medical Sciences, The University of Aberdeen, Aberdeen, UK

**Keywords:** delirium, ICU, QEEG, EEG, Granger causality, coherence, connectivity

## Abstract

Delirium is an under-diagnosed yet frequently occurring clinical complication with potentially serious consequences for intensive care unit (ICU) patients. Diagnosis is currently reactive and based upon qualitative assessment of the patient's cognitive status by ICU staff. Here, we conducted a preliminary investigation into whether emerging quantitative electroencephalography (QEEG) analysis techniques can accurately discriminate between delirious and non-delirious patients in an ICU setting. Resting EEG recordings from 5 ICU patients in a state of delirium and 5 age matched control patients were analyzed using autoregressive spectral estimation for quantification of EEG power and renormalized partial directed coherence for analysis of directed functional connectivity. Delirious subjects exhibited pronounced EEG slowing as well as severe general loss of directed functional connectivity between recording sites. Distinction between groups based on these parameters was surprisingly clear given the low sample size employed. Furthermore, by targeting the electrode positions where effects were most apparent it was possible to clearly segregate patients using only 3 scalp electrodes. These findings indicate that quantitative diagnosis and monitoring of delirium is not only possible using emerging QEEG methods but is also accomplishable using very low-density electrode systems.

## 1. Introduction

Delirium is an acute state of confusion that is common in intensive care unit (ICU) patients. The diagnostic and statistical manual of mental disorders–version 5 (DSM-V) states that the core symptoms encompass reduced attention, impaired memory and cognition, language disturbance and lack of orientation to time and space [1]. Delirium is currently under-diagnosed and poorly managed. indeed, the condition has historically been considered an almost inevitable component of critical care and many specialists considered the condition to be transitory and of little clinical significance [2]. Consequently, only 6.4% of critical care professionals routinely use specific assessment tools to monitor for the condition [3]. This is despite research demonstrating numerous negative clinical outcomes associated with delirium including increased mortality, longer duration of hospital stay, increased costs and long-term cognitive impairment [4].

Sedation levels and endotracheal intubation limit a patient's ability to communicate, thus hindering a definitive diagnosis. To combat this, ICU specific delirium screening tools have been developed. Guidelines from the National Institute for Health and Care Excellence (NICE) currently recommend “Confusion Assessment Method for the Intensive Care Unit” (CAM-ICU) screening [5],[6]. In practical terms, the CAM-ICU screens for delirium by assessing for an acute change or a fluctuating course of mental status. If positive, the patient is formally assessed for inattention, altered consciousness and disorganized thinking. The CAM-ICU has been reported to have a sensitivity of 93–100% and specificity of 89–100% in research settings [5],[7]. However, in a routine clinical setting, while specificity remains high, sensitivity of the CAM-ICU is substantially reduced (47%), with sensitivity being particularly poor for hypoactive delirium (31%) [8]. This issue alongside the fact that many critical care specialists fail to regularly screen for delirium indicate than an alternative, sensitive screening approach is needed.

Studies using quantitative electroencephalography (QEEG) techniques have previously reported a “slowing” of the EEG in delirious subjects [9]–[11]. Specifically, delirium is associated with increased Theta and Delta power with reduced Alpha power [10]. Furthermore, the magnitude of EEG slowing correlates with cognitive impairment and abates with recovery [12]. These findings raise the possibility that QEEG techniques may offer a route towards quantitative detection and monitoring of delirium. Interestingly, Gamma [> 20 Hz] activity, a feature of high-level brain function, has largely been neglected in ICU QEEG studies, likely due to the low relative amplitude of the Gamma band and potential contamination with muscular activity occurring at similar frequencies [13]. Additionally, there is inherent difficulty in obtaining good quality EEG recordings in delirious patients who often struggle following instructions to (for example) remain stationary with their eyes closed for an extended period of time [14]. The adoption of auto-regressive (AR) spectral estimation, which offers a significant enhancement of signal-to-noise ratio over standard Fast Fourier Transform (FFT) based spectral analyses [15] may therefore be advantageous.

Reduced functional connectivity in delirium has previously been reported using resting state functional magnetic resonance imaging [16]. Very few studies have investigated EEG functional connectivity in delirium. Those which have employed EEG have generally reported a loss of inter-regional connectivity, similar to the effect of anesthesia [14],[17]. Renormalized partial directed coherence (rPDC) is a recently reported [18],[19] addition to the Granger causality [20] based family of brain connectivity estimators. Briefly, rPDC utilizes multivariate autoregression as a measure of Granger causality, in order to investigate causal (rather than correlational) relationships between EEG activity recorded at two or more electrode sites. By doing so it is possible to determine the frequency and strength of connectivity among channel pairs for both directions independently (Eg C3-T3 vs T3-C3). This is possible even when the source and sink of neural signals communicate indirectly via an intermediate node (Eg, P3-C3-T3). Similar to the underlying AR analyses, rPDC is well suited to noisy data [19] and has recently been proven highly effective in preclinical research applications [15] but has not yet been applied to the study of delirium.

Here, we present the findings of a pilot study aiming to evaluate the potential utility of AR-power and rPDC-connectivity analyses for identification of delirium in an ICU setting. In brief, using only a sparse electrode configuration, both pronounced EEG slowing and greatly reduced directed functional connectivity were observed in delirious subjects. Our findings are compared with those of more traditional QEEG approaches, and recommendations are made for future studies.

## 2. Methods

### 2.1. Study design

Ethical approval was granted by the “Scottish Research Ethics Committee (REC)” (REC number–15/SS/0214) and research and development approval was attained for NHS Grampian. The study was conducted at the ICU in Aberdeen Royal Infirmary (ARI); patients admitted to the ICU were screened for study eligibility over a 2 months period. As the study was observational, no patient treatment was altered for the purpose of the study, the only change in a patients care following enrolment was an EEG recording session. Study personnel were not blinded and patients were not randomized. Written informed consent was obtained from all patients or when appropriate, from a legal representative of the patient; a photocopy of the consent form was placed in their notes to indicate enrolment in the study.

### 2.2. Subjects

59 patients were pre-screened for entry to the study upon admission to the ICU, 5 of which were excluded outright. Patients were excluded at pre-screening if they were currently pregnant or under 16 years old. Those with pre-existing conditions of cognitive decline, such as dementia, were excluded as such conditions may confound the diagnosis of delirium. To ensure enrolled patients were physically able to undergo an EEG recording session, those with head injuries were excluded. Patients with conditions known to be associated with disrupted EEG patterns, such as epilepsy and psychiatric conditions, were also excluded. A CAM-ICU assessment was performed daily, and patients were eligible for inclusion in the delirious group if they had a positive CAM-ICU score and a Richmond Agitation- Sedation Score (RASS) of −3 or greater. A RASS > = −3 requires movement in response to voice indicating that the patient is not unconscious and capable of muscular movement. Control subjects were also recruited from ICU patients to ensure a comparable severity of baseline illness. Reasons for admission to the ICU are provided for each patient in supplementary Table 1. Inclusion criteria for control patients included a negative CAM-ICU and a RASS of 0 indicating the patient was alert and responsive. Of the 54 patients who passed initial screening, 14 were identified as delirious and 15 were eligible to be control subjects, the rest failed to fulfil the inclusion criteria for either group. Written informed consent was obtained from legal representatives for 7 delirium subjects. For the control patients, all 7 control subjects retained capacity and therefore provided written informed consent themselves. EEG data recorded from 2 delirium and 2 control subjects were excluded during the analysis stage due to 1 case of a technical problems and 3 cases of heavy electrical artefact contamination. The average age of included subjects was similar between groups, however, there were more males in the non-delirium group (see Table 1). All centrally acting psychoactive medications administered within the 24 hours preceding the EEG recording session were documented.

**Table 1.** Study cohort: Age, gender and medication status of study cohort.

	Delirium Subjects (n = 5)	Non-Delirium Subjects (n = 5)
Age, mean (range)	62	65.6
Gender: male, n (%)	3 (60%)	5 (100%)
Sedative Medication (Within 24 hours of recording)		
Propofol (%)	0	1 (20%)
Alfentanil (%)	2 (40%)	1 (20%)
Morphine (%)	1 (20%)	0
Oxycodone (%)	1 (20%)	0

### 2.3. EEG recordings

EEG recordings were made as soon as possible following discovery of eligible subjects. The CAM-ICU assessment was then repeated immediately before start of recording. Patients were fitted with an elastic head-cap with sockets for superficial recording electrodes situated according to the 10-20 system. Conductive gel (Sigma gel, Parker Laboratories Inc, USA) was applied to the scalp using a syringe placed into each socket. Recording electrodes were then placed at bilateral frontal (F3 + F4), Central (C3, C4) and parietal (P3, P4) and temporal (T7, T8) positions. Recording electrodes were referenced to a common mode sense electrode placed at position C2. Recordings were conducted using the ActiveTwo EEG system, and Actiview (v8.06) acquisition software (BioSemi, NL); data were digitized at 2048 Hz, band pass filtered (high pass = 0.16 Hz, low pass = 52 Hz) and then down-sampled to 256 Hz. EEG activity was recorded for approximately 10 minutes with the patient supine and instructed to keep eyes closed. Understandably, compliance with these requirements was not absolute among delirious patients, therefore, times of unexpected eye-opening and/or movement were noted at time of recording and avoided during analysis. Three 60-second samples of continuous EEG devoid of major artefacts were then identified through visual inspection of the raw EEG trace and extracted.

### 2.4. EEG analysis

AR spectral estimation and rPDC connectivity analyses were conducted as described previously [15] using custom written MATLAB scripts (Mathworks Inc, USA). Briefly, AR power spectra with 0.5 Hz frequency resolution were calculated for each sample using an AR order of 64 (sampling rate/4). AR spectra were then averaged within subjects to derive subject average spectra (n = 5 per group). EEG frequency bands were defined as; Delta = 0.4–4 Hz, Theta = 4–8 Hz, Alpha = 8–13 Hz, Beta = 13–20 Hz and Gamma = 20–45 Hz.

Absolute AR power measured at all frequencies points within each frequency band were summed in order to reduce the power spectrum to a set of band power values. These were then converted to relative power values prior to analysis. Given the high relative power of Delta waves (approx. 80% of total signal energy), and that movement artefacts generate erroneous power predominantly in the Delta range, relative Delta power was quantified separately from other bands. Delta band power was expressed as a percentage of the summed power of all frequency points in the 0.5–45 Hz range. All other band powers were expressed as % summed power between 4 and 45 Hz. This segregation ensured that relatively small changes in relative Delta power due to movement artefacts would not confound the interpretation of relative power changes at higher frequencies.

Non-matching 2-way ANOVAs (α = 0.05) with patient status (delirium vs control) and recording channel as factors were then performed for each EEG band. Where a significant effect of channel or interaction was observed, Bonferroni post-tests were deployed to compare relative power between groups at individual electrode sites. However, it must be conceded that while the sample size used in this exploratory study is sufficient to determine effect sizes, estimates of statistical significance must be treated with appropriate caution.

In directed connectivity analysis among *n* channels, there are *n*(*n*−1) possible communication directions. Therefore, to avoid excessive complexity, rPDC analysis was conducted on a left hemispheric channel sub-set (F3, C3, P3, and T7) in order to represent all regions in the analysis. Frequency and direction resolved rPDC strength was calculated for each EEG sample and pooled (n = 15 per group). Statistical comparison of rPDC strength was conducted for each EEG band as well as across the entire 0.5-45 Hz range for each channel pair and direction. A significant difference exists at a single frequency point where the mean of one group lies outside the 95% confidence interval of the other. A significant difference between groups over a frequency range therefore exists when the number of points at which groups significantly differ exceeds what is predicted at random.

## 3. Results

### 3.1. Auto-regressive spectral analysis

**Figure 1.**
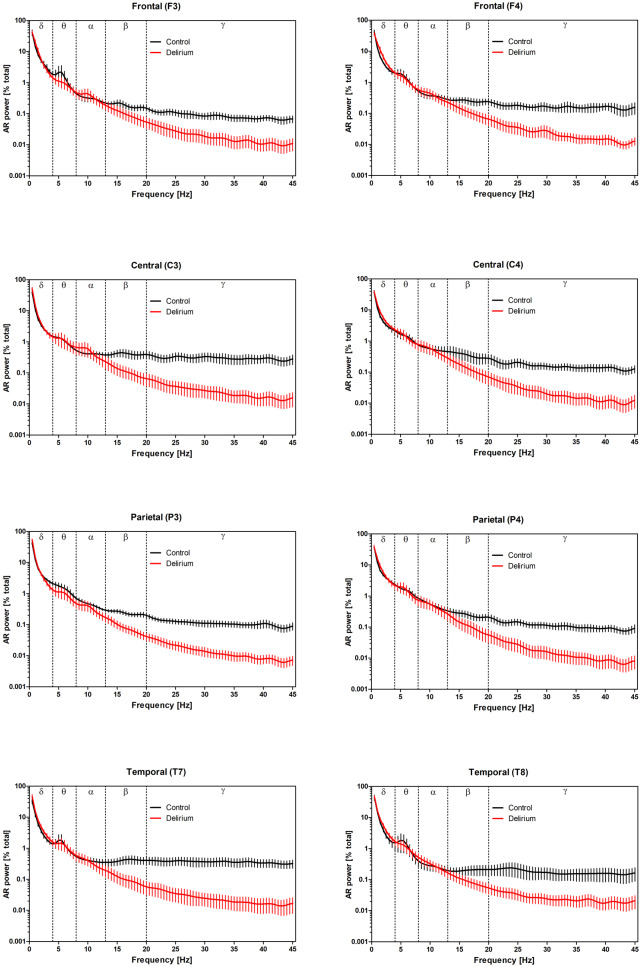
Relative power spectra of delirious and non-delirious subjects. Mean ± SEM AR power spectral estimates for control (black) and delirium (red) groups (normalized to total power) are presented for all 8 recording channels. Dashed vertical lines delineate cut-offs between Delta (δ), Theta (θ), Alpha (α), Beta (β) and Gamma (γ) bands.

AR power spectra obtained from all 8 recording channels, normalized relative to total power (summed power at frequencies from 0.5–45 Hz) are presented in Figure 1. Cursory examination of the plots indicates a substantial reduction in Beta and Gamma power in delirious subjects across all channels.

**Figure 2.**
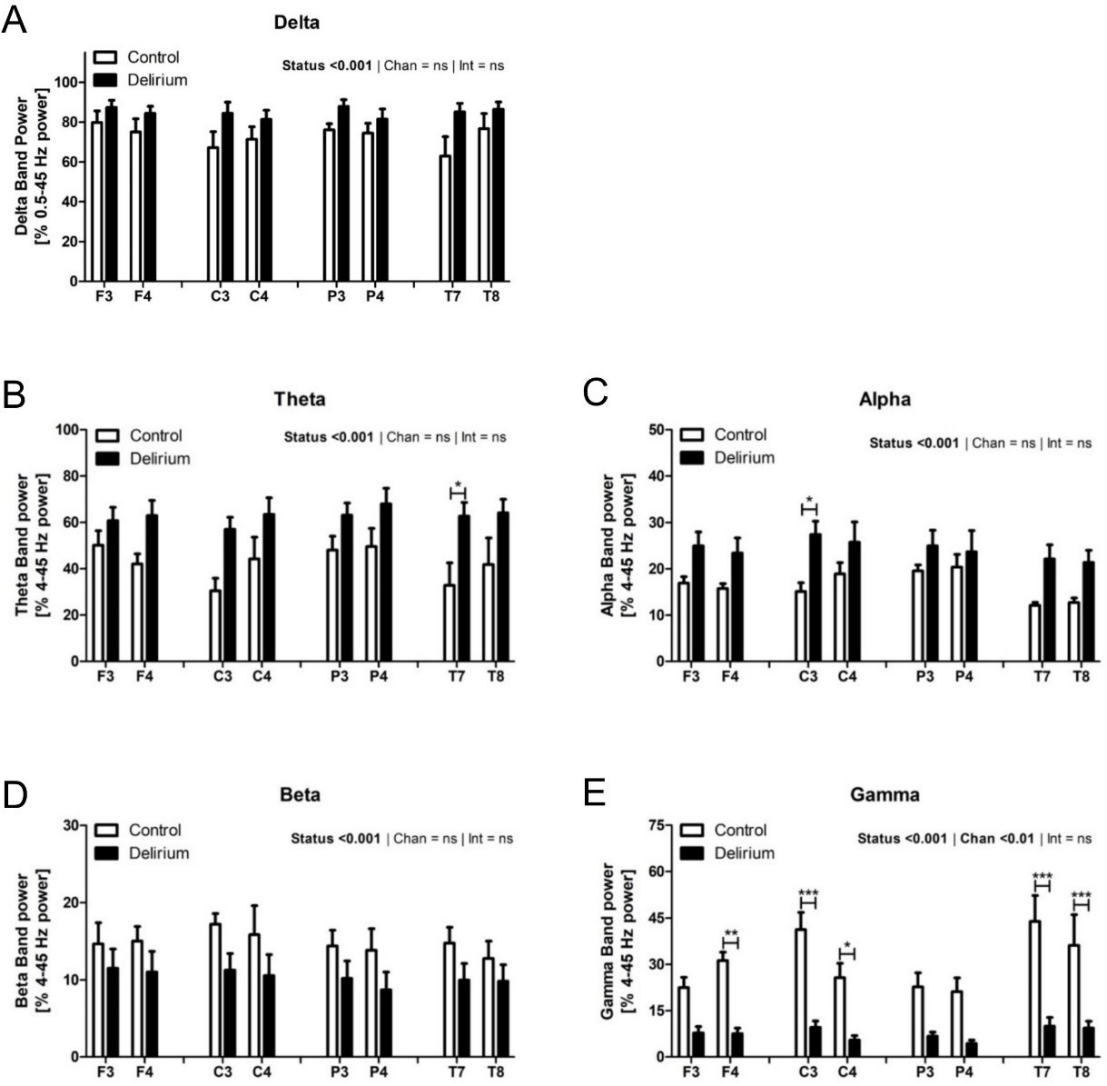
Delta, Theta and Alpha power increased while Beta and Gamma decreased in delirium. A: Delta band (0.5–4 Hz) power (mean ± SEM as % total 0.5–45 Hz power) for control (white) and delirium (black) groups. B: Theta (4–8Hz), C: Alpha (8–13 Hz), D: Beta (13–20 Hz) and E: Gamma (20–45 Hz) band power (means ± SEM as % total 4–45 Hz power) for control (white) and delirium (black) patient groups. Data are presented for left and right frontal (F3, F4), central (C3,C4), parietal (P3, P4) and temporal (T7, T8) recording channels. Significances are indicated for effects of delirium vs control (status), recording channel (Chan) and interaction (Int). Asterisks above horizontal bars indicate results of Bonferroni post-tests comparing groups at each electrode location. * = p < 0.05, ** = p < 0.01, *** = p < 0.001.

Analysis of summed Delta band power relative to total (0.5–45 Hz) power (Figure 2A) indicated significantly higher Delta power content in the EEG of delirious patients [F(1,64) = 17.43, p < 0.001] with no effects of either channel or interaction [F < 1]. Theta to Gamma bands were determined relative to total power between 4 and 45 Hz (Figure 2 B–E). Relative to controls, delirious patients demonstrated globally enhanced Theta [Status: F(1,64) = 33.17, p < 0.001, Channel: F < 1, Interaction: F < 1] and Alpha power [Status: F(1,64) = 32.62, p < 0.001 Channel: F(7,64) = 1.281, p > 0.05 Interaction: F < 1]. Conversely, Beta power was reduced in delirious subjects [Status: F(1,64) = 13.40, p < 0.001, Channel: F < 1, Interaction: F < 1]. Gamma power was also generally reduced in delirium [F(1,64) = 108.5, p < 0.001] and the effect of recording channel was also found to be significant [F(7,64) = 2.964, p < 0.01] with no significant interaction [F < 1]. Subsequent Bonferroni post-tests detected significant differences between patient groups for channels F4, C3, C4, T7 and T8. Despite the increased variability of absolute vs relative power estimates, similar changes were visible in absolute power spectra (supplementary Figure 1) indicating that these effects were not attributable to an artefact of conversion to absolute power.

### 3.2. Connectivity analysis (rPDC)

**Figure 3.**
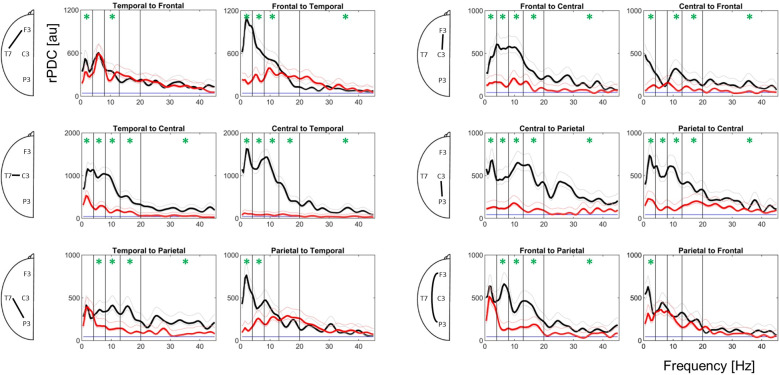
Reduced directed connectivity in delirious subjects. Strength of directed coherence is presented for all possible combinations of left hemisphere channels and directions of communication. The frequency [Hz] of signals are given by the x-axes while rPDC communication strength [arbitrary units] are given by the y-axes. Thick coloured lines indicate delirium (red) and control (black) group means. Thin coloured lines depict the 95% confidence interval of associated group means. Blue horizontal lines indicate the level of rPDC strength expected by chance. Forward and reverse directions of communication are paired by pathway (e.g. top left pair = temporal to frontal vs frontal to temporal). Half-head diagrams on the left indicate the location of channels analysed. Vertical lines delineate frequency band limits. Asterisks indicate significant differences between groups for the associated band.

Plots of frequency and direction-resolved connectivity strength among all possible pairings of left hemisphere recording sites are presented in Figure 3. Statistical comparison between patient groups across the entire 0.5–45 Hz range indicated significantly reduced connectivity in delirious patients for all combinations and directions with the exception of parietal to frontal communication (frontal to parietal impaired). Subsequent comparison of individual bands (see asterisks in Figure 3) revealed that central-parietal and temporal-central connectivity were most severely reduced in delirium. Bi- directional reductions in connectivity among these pairings were observed at all frequency bands. The same was largely true of central-frontal connectivity with the exception of the Theta band specifically in the central to frontal direction. All other combinations demonstrated some degree of band and frequency specificity cf. the loss of communication in delirious subjects. Interestingly, there was an apparent relative sparing of parietal to frontal connectivity where only Delta connectivity was altered in delirium. Similarly, temporal to frontal connectivity was impaired in only Delta and Alpha bands. Therefore, while delirious patients exhibit a global loss of functional connectivity, the central-temporal and central-parietal pairings appear most robust to distinguish between delirious subjects.

### 3.3. Optimised AR and rPDC criteria

**Figure 4.**
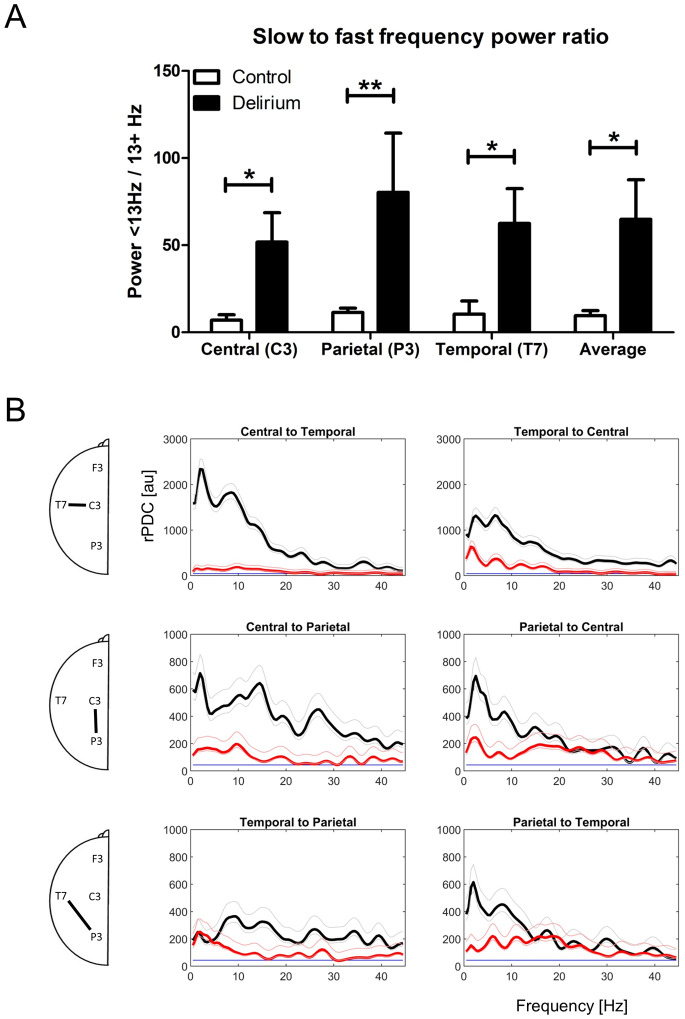
Optimized QEEG analysis. A: Slow (< 13 Hz) to fast (13+ Hz) frequency power ratio (mean ± SEM) of EEG recorded at selected central, parietal and temporal electrode positions in delirious subjects (black) and healthy controls (white). The average power ratio across all 3 sites is also presented. Asterisks indicate p values of non- parametric tests, * = p < 0.05, ** = p < 0.01. B: Strength of directed coherence (rPDC, arbitrary units) is presented for all possible connections among the C3 (central), P3 (parietal) and T7 (temporal) electrodes. Diagrams to the left of pairs indicate location of channels analysed in each row.

The above results indicate that while the magnitude of EEG power and connectivity changes vary by electrode the observed effects are global in nature. This raises the possibility that by targeting the electrodes with greatest differences, discrimination between groups may be accomplished using a smaller number of electrodes. This is particularly relevant for ICU settings where reducing numbers of electrodes increases the feasibility of performing EEG under challenging conditions. The analytic readout can also be simplified substantially. To evaluate this possibility, a slow-to-fast frequency power ratio (SFPR) was calculated based on the summed absolute power of all frequencies below 13 Hz divided by that of frequencies from 13–45 Hz for the C3, P3 and T7 electrode positions. Comparison of channel SFPRs between groups via 2-tailed Mann-Whitney tests confirmed that SFPRs provide a robust distinction between patient groups, i.e. delirious subjects expressed a significantly higher SFPR than healthy controls (Figure 4 A) for each individual channel, C3 [p < 0.05], P3 [p < 0.01], T7 [p < 0.05]. This was also true when SFPRs were averaged across all 3 channels [p < 0.05]. EEG power changes in delirium, as measured using AR can therefore be detected using a small number of electrodes and quantified as a single value - the averaged SFPR of all electrodes.

Because rPDC analysis considers indirect as well as direct connections, connectivity strength estimates for any given connection naturally vary when channels are removed from the model. It is important therefore to consider whether rPDC results observed for a particular connection are reproducible when the electrode configuration is pared down to only a subset of interest. Therefore, the rPDC analysis was repeated using only the three channels used in the above AR analysis. As connectivity among these channels was universally decreased at all frequencies, comparison between groups was performed over the entire 0.5–45 Hz range rather than for individual bands. This analysis (Figure 4B) confirmed the significant reduction in connectivity strength for all possible channel pairs and communication directions. Therefore, the finding of reduced connectivity under delirium is reproducable using only 3 electrodes and comparison over the full EEG frequency range rather than for discrete bands individually.

## 4. Discussion

Our observation of generally decreased connectivity among brain regions in delirious subjects lends evidence to the concept of delirium as a “disconnection syndrome” [14]. However, in part due to the complex aetiology of delirium, the precise neurological changes underlying this loss of connectivity remain poorly understood. We suggest that reversible functional disintegration of critical network hubs in the association cortices give rise to delirium. Here, we shall discuss the evidence in support of this viewpoint, examine potential confounding factors in this study and offer guidance for future research.

### 4.1. Directed connectivity in delirium

Studies of directed connectivity based on directed phase lag index (dPLI) have indicated that activity in frontal regions phase leads that of parietal and occipital regions in healthy resting subjects. Mathematical models have predicted that major hubs of the default mode network located in the parietal regions may act as information attractors or “sinks” [21]. The dominant front-to-back pattern of information flow could therefore emerge as a natural consequence of default mode network function and reflect a flow of information from anterior brain regions to parietal integration centres. Of particular note is the observation that normal anterior to posterior gradient of Delta band connectivity is reversed in delirium following cardiac surgery [14]. This suggests that post-operative delirium may result from functional collapse of parietal integration centres. Interestingly, a second study using phase transfer entropy (PTE) rather than dPLI as a measure of directed connectivity indicated that alpha band information predominantly flows in the posterior to anterior direction and this is abolished under delirium [17]. Therefore, while altered direction of information flow appears to be a common finding in delirium, there is some disagreement among connectivity analysis techniques regarding the typically dominant direction. This may be due to the fact that dominant direction is affected by reference location in the case of dPLI [22] and frequency in the case of PTE [23].

While the rPDC approach deployed here differs from analyses used in previous studies our findings are congruent with evidence for parietal disintegration in delirium. We observed a severe disruption of frontal to parietal connectivity in delirious subjects with an apparent sparing of parietal to frontal connectivity (Figure 3). Similarly, Theta connectivity was impaired in the frontal to central direction but not in the reverse direction under delirium. While the disruption of communication under delirium was less frequency-specific than reported by Van Dellen, et al (2014), these observations remain consistent with more severe suppression of anterior to posterior information flow. Furthermore, when the frontal recording site was excluded from analysis (Figure 4) our findings indicate a severe impairment of communication for all channel pairs. This implies a concentration of dysfunction in the posterior region.

### 4.2. EEG and sedative medications in the ICU

Reversal of the dominant direction of information flow, similar to that described here is also observed under propofol and sevoflurane [24],[25]. This is thought to arise from a more severe impact of anaesthetic agents on information processing in the parietal vs frontal networks [26]. This could result in frontal hubs becoming the major information attractors in the default network. It has therefore been argued that disruption of information processing and integration due to suppression of Gamma activity and network disintegration represents a common route to unconsciousness under general anaesthesia [24],[27]. Similarly, sub-anaesthetic doses of anti-GABAergic sedatives such as those commonly administered to ICU patients, are known to alter EEG coherence and high frequency activity [28],[29]. This does raise the question of whether the effects observed here in delirious subjects may result partially from the effect of centrally acting sedatives. However, while the limited scope of this study precludes a detailed analysis of drug effects, similar proportions of delirious and non-delirious patients had received propofol / alfentanil and EEG alterations noted here were only apparent in the delirious group. It is also noteworthy that use of propofol its-self may be a factor which enhances the risk of post-operative delirium [30]. Dissecting the influences of common ICU medications on neural network function will require a larger controlled study controlling for medication status. However, we propose that the observable neuro-cognitive symptoms of delirium, upon which diagnosis is currently based, may result from the abnormal adoption of a neural network state in awake individuals similar to that which occurs under light anaesthesia. This may occur in response to any number of physical or chemical insults, including administration of e.g. sedatives in susceptible individuals.

### 4.3. Effect of age and neurological disorder

The patient groups examined in this study were quite well balanced in terms of age and patients with head injuries or known psychiatric illnesses were excluded from participation. These factors were therefore unlikely to contribute to group differences in EEG readouts observed here. However, in a real-world diagnostic application, broad variation in age and physical / mental health among patients would be common. It is therefore important to consider whether the EEG signatures of delirium are sufficiently sensitive, selective and robust in the presence of these confounders. For example, EEG slowing as observed here with AR analysis, is associated with normal ageing [31], and is further exacerbated in dementia [32]. Dementia is also associated with reduced EEG connectivity and reorganization of network hubs [33]. The expected values of SFPRs and connectivity strength may therefore require calibration according to a patient's health status in order to adequately discern the additional effect of delirium. A larger scale study recruiting a more representative cross-section of ICU patients would allow the effect of common conditions and medication effects on AR and rPDC readings to be modelled allowing for this adjustment. An alternative approach would be to adopt a study protocol where resting EEG is continuously recorded over a long duration. Parameters quantifying the within-subject variability regional power and inter-regional connectivity could then potentially be interrogated for viable biomarkers as an alternative to comparison against a control group.

### 4.4. CAM-ICU for the assessment of delirium

Previous studies deployed teams of “delirium experts”, composed of geriatricians, neurologists and psychiatrists, to confirm the diagnosis of delirium following an initial CAM-ICU screening. A potential criticism of the present study may be the exclusive use of the CAM-ICU to identify delirious subjects. As delirium fluctuates over time CAM-ICU assessments were repeated here immediately prior to a recording session to ensure (as far as possible) the patient was deemed to be delirious during data capture. Reliance on teams of experts for the assessment of patients immediately prior to EEG recording sessions is neither practical nor achievable in a real-world application. However, in a larger validation study it would certainly be advisable to recruit an expert monitoring team for the purpose of both improving confidence in allocation of patient groups and continuous assessment of delirium status to parallel continuous monitoring of EEG.

### 4.5. Future adaptions of recording procedures

The EEG sampling procedure here was intended to capture a “snapshot” of EEG activity immediately following identification of delirium. We therefore utilized relatively long EEG epochs during which the patient's condition was considered invariant. However, one of the major advantages of AR and rPDC vs traditional approaches is that they allow excellent resolution of both amplitude and frequency of EEG activity even on very small time scales [15]. In future, it is recommended to reduce the epoch size to 1–2 seconds and chart the within-subject variability of key EEG parameters in order to measure the stability of brain networks as an additional parameter. Additionally, due to the impaired ability of some ICU patients to comply with instructions (such as to open or close eyes) resting state EEG parameters requiring patients to follow a specific command may be highly variable. It would therefore be interesting to also examine involuntary EEG responses to sensory stimuli such as presentation of a tone. The time-varying rPDC approach [15] could then be used to examine brain network responses on millisecond time scales. Finally, similar to previous studies [34] we have demonstrated that QEEG parameters retain high discriminative power using only sparse electrode configurations. However, by recording only from the most dysfunctional network nodes we may retain sensitivity but sacrifice the specificity that may come from examining connections left intact under delirium, but not other conditions with diagnostic overlap. We recommend that future exploratory studies should still utilize a higher electrode density so that the minimal electrode configuration required to achieve suitable sensitivity and specificity can be determined.

## 5. Conclusions

Within the limitations of this pilot study, AR and rPDC analysis distinguished robustly between delirious and non-delirious ICU patients. Future studies are required to explore the impact of other confounders, for example age, medication and health status. However, given the strong performance of these methods on noisy signals and the large apparent magnitude of EEG alterations under delirium, we propose that AR and rPDC carry significant potential for QEEG based detection of delirium in a challenging recording environment.

## References

[1] American Psychiatric Association (APA) (2013). Diagnostic and statistical manual of mental disorders (5th ed.).

[2] Truman B, Ely EW (2003). Monitoring delirium in critically ill patients. Using the confusion assessment method for the intensive care unit. Crit Care Nurse.

[3] Ely EW, Stephens RK, Jackson JC (2004). Current opinions regarding the importance, diagnosis, and management of delirium in the intensive care unit: A survey of 912 healthcare professionals. Crit Care Med.

[4] Boot R (2012). Delirium: A review of the nurses role in the intensive care unit. Intensive Crit Care Nurs.

[5] Ely EW, Margolin R, Francis J (2001). Evaluation of delirium in critically ill patients: Validation of the Confusion Assessment Method for the intensive care unit (CAM-ICU). Crit Care Med.

[6] Ely EW, Shintani A, Truman B (2004). Delirium as a predictor of mortality in mechanically ventilated patients in the intensive care unit. J Am Med Assoc.

[7] Han JH, Wilson A, Graves AJ (2014). Validation of the confusion assessment method for the intensive care unit in older emergency department patients. Acad Emerg Med.

[8] Van Eijk MM, Van Den Boogaard M, Van Marum RJ (2011). Routine use of the confusion assessment method for the intensive care unit: A multicenter study. Am J Respir Crit Care Med.

[9] Jacobson S, Jerrier H (2000). EEG in delirium. Semin Clin Neuropsychiatry.

[10] Shafi MM, Santarnecchi E, Fong TG (2017). Advancing the neurophysiological understanding of delirium. J Am Geriatr Soc.

[11] Koponen H, Partanen J, Pääkkönen A (1989). EEG spectral analysis in delirium. J Neurol Neurosurg Psychiatry.

[12] Jacobson SA, Leuchter AF, Walter DO (1993). Serial quantitative EEG among elderly subjects with delirium. Biol Psychiatry.

[13] Whitham EM, Pope KJ, Fitzgibbon SP (2007). Scalp electrical recording during paralysis: Quantitative evidence that EEG frequencies above 20 Hz are contaminated by EMG. Clin Neurophysiol.

[14] Van Dellen E, Van Der Kooi AW, Numan T (2014). Decreased functional connectivity and disturbed directionality of information flow in the electroencephalography of intensive care unit patients with delirium after cardiac surgery. Anesthesiology.

[15] Crouch B, Sommerlade L, Veselcic P (2018). Detection of time-, frequency- and direction-resolved communication within brain networks. Sci Rep.

[16] Choi SH, Lee H, Chung TS (2012). Neural network functional connectivity during and after an episode of delirium. Am J Psychiatry.

[17] Numan T, Slooter AJC, van der Kooi AW (2017). Functional connectivity and network analysis during hypoactive delirium and recovery from anesthesia. Clin Neurophysiol.

[18] Schelter B, Timmer J, Eichler M (2009). Assessing the strength of directed influences among neural signals using renormalized partial directed coherence. J Neurosci Methods.

[19] Sommerlade L, Thiel M, Mader M (2015). Assessing the strength of directed influences among neural signals: An approach to noisy data. J Neurosci Methods.

[20] Granger CWJ (1969). Investigating causal relations by econometric models and cross-spectral methods. Econometrica.

[21] Stam CJ, van Straaten ECW (2012). Go with the flow: Use of a directed phase lag index (dPLI) to characterize patterns of phase relations in a large-scale model of brain dynamics. Neuroimage.

[22] Guevara R, Velazquez JLP, Nenadovic V (2005). Phase synchronization measurements using electroencephalographic recordings: What can we really say about neuronal synchrony?. Neuroinformatics.

[23] Hillebrand A, Tewarie P, van Dellen E (2016). Direction of information flow in large-scale resting-state networks is frequency-dependent. Proc Natl Acad Sci.

[24] Lee H, Mashour GA, Noh GJ (2013). Reconfiguration of network hub structure after propofol-induced unconsciousness. Anesthesiology.

[25] Ku SW, Lee U, Noh GJ (2011). Preferential inhibition of frontal-to-parietal feedback connectivity is a neurophysiologic correlate of general anesthesia in surgical patients. PLoS One.

[26] Lee U, Müller M, Noh GJ (2011). Dissociable network properties of anesthetic state transitions. Anesthesiology.

[27] Hudetz A, Hudetz A, Pearce R (2009). Cortical disintegration mechanism of anesthetic-induced unconsciousness. Suppressing the Mind.

[28] Pal D, Silverstein BH, Sharba L (2017). Propofol, sevoflurane, and ketamine induce a reversible increase in Delta-Gamma and Theta-Gamma phase-amplitude coupling in frontal cortex of rat. Front Syst Neurosci.

[29] Seifert HA, Blouin RT, Conard PF (1993). Sedative doses of propofol increase beta activity of the processed electroencephalogram. Anesth Analg.

[30] Brown KE, Mirrakhimov AE, Yeddula K (2013). Propofol and the risk of delirium: exploring the anticholinergic properties of propofol. Med Hypotheses.

[31] Klimesch W (1999). EEG alpha and theta oscillations reflect cognitive and memory performance: A review and analysis. Brain Res Rev.

[32] Cassani R, Estarellas M, San-Martin R (2018). Systematic review on resting-state EEG for Alzheimer's disease diagnosis and progression assessment. Dis Markers.

[33] Engels MMA, Stam CJ, van der Flier WM (2015). Declining functional connectivity and changing hub locations in Alzheimer's disease: An EEG study. BMC Neurol.

[34] van der Kooi AW, Zaal IJ, Klijn FA (2015). Delirium detection using EEG. Chest.

