# Supplementary material for: Delirium screening in the intensive care unit using emerging QEEG techniques: A pilot study
Source: AIMS Neurosci. 2020 Jan 13;7(1):1–16. doi: 10.3934/Neuroscience.2020001 (PMC7242058; doi:10.3934/Neuroscience.2020001)
Supplement: Supplementary file 1 [file neurosci-07-01-001-s001.pdf]

AIMS Neuroscience

http://www.aimspress.com/journal/neuroscience

AIMS Neuroscience, 7(1): 1–16.
DOI: 10.3934/Neuroscience.2020001
Received: 15 October 2019
Accepted: 05 January 2020
Published: 13 January 2020

*Research article*

# Delirium screening in the intensive care unit using emerging QEEG techniques: A pilot study

**Andrew Hunter, Barry Crouch, Nigel Webster and Bettina Platt***

Institute of Medical Sciences, The University of Aberdeen, Aberdeen, UK

* **Correspondence:** Email: B.Platt@abdn.ac.uk; Tel: +44 (0)1224 437402.

**Supplementary Table 1.** Study cohort—Reasons for ICU admission.

| Control group | | Delirium group | |
|---|---|---|---|
| C1 | Septic shock | D1 | Bilateral pneumonia |
| C2 | Worsening hypoxaemia | D2 | Pneumonia |
| C3 | Pulmonary oedema | D3 | Sepsis |
| C4 | Post-operative care (laparotomy) | D4 | PUJ* obstruction & hydronephrosis |
| C5 | Type 1 respiratory failure | D5 | High $O_2$ requirements & ionotrope therapy |

*Note: Pelvic Ureteric Junction.

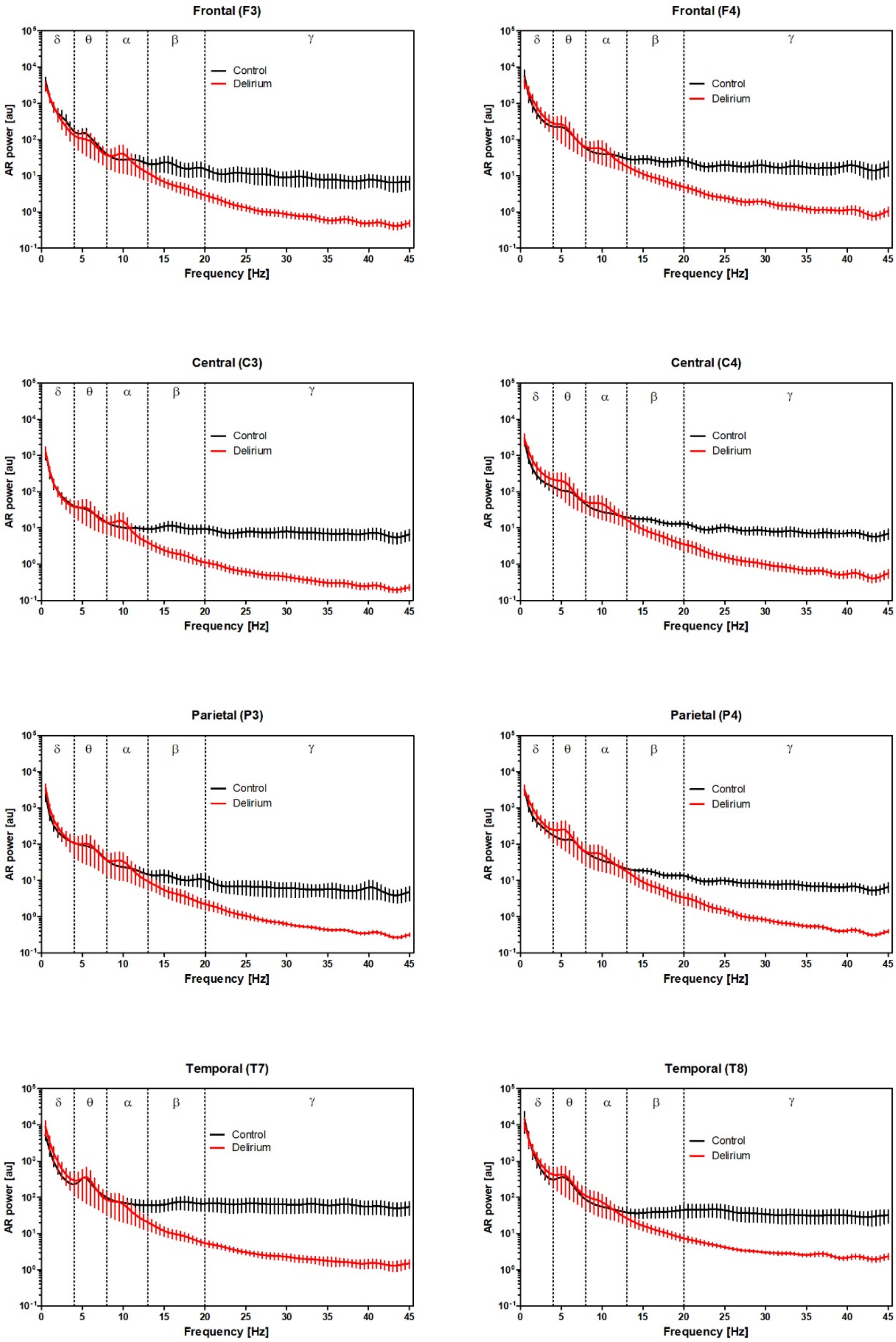


**Supplementary Figure 1.** Absolute power spectra of delirious and non-delirious subjects. Mean ± SEM AR power (arbitrary units) spectral estimates for control (black) and delirium (red) groups are presented for all 8 recording channels. Dashed vertical lines delineate cut-offs between Delta (δ), Theta (θ), Alpha (α), Beta (β) and Gamma (γ) bands.

AIMS Press

© 2020 the Author(s), licensee AIMS Press. This is an open access article distributed under the terms of the Creative Commons Attribution License (http://creativecommons.org/licenses/by/4.0)
